# Associations of palmar surface lactic acid with hand scaling and *in vitro* pathogen inactivation: A cross-sectional study

**DOI:** 10.1371/journal.pone.0362451

**Published:** 2026-11-10

**Authors:** Kenichi Nagano, Kenji Manabe, Ichiro Mori, Yasuhiro Okada, Eri Shimizu, Satoru Naito

**Affiliations:** 1 Human Health Care Products Research, Kao Corporation, Tokyo, Japan; 2 Analytical Science Research, Kao Corporation, Tokyo, Japan; University of Manchester School of Biological Science: The University of Manchester Faculty of Biology Medicine and Health, UNITED KINGDOM OF GREAT BRITAIN AND NORTHERN IRELAND

## Abstract

The relationship between palmar surface lactic acid and hand scaling is not well understood. This cross-sectional study examined associations between palmar surface lactic acid, hand scaling, and *in vitro* anti-pathogenic activity, to generate hypotheses relevant to hand skin condition and pathogen inactivation. In 49 healthy adults, after standardized hand washing and acclimation (20°C, 40% RH), we assessed scaling (visual score), surface lactic acid and stratum corneum ceramides (LC–MS), stratum corneum amino acids (ion-exchange chromatography), skin pH and temperature, and sweat rate. Anti-pathogenic activity of palmar extracts was quantified as log_10_ reduction against influenza A/H3N2, *Escherichia coli* NBRC3301, and *Staphylococcus aureus* NBRC13276. Associations were evaluated using Spearman and partial correlations with Benjamini–Hochberg correction for false discovery rate. Higher lactic acid content was associated with less scaling. Stronger anti-pathogenic activity was also associated with less scaling. Total ceramides and total amino acids were not significantly associated with scaling, whereas several ceramide classes showed negative associations with scaling. In partial correlation models, lactic acid remained independently associated with greater anti-pathogenic activity and with lower scaling (significant in two models and trend-level in one model), and the simple correlation between anti-pathogenic activity and scaling was attenuated after accounting for lactic acid. Lactic acid was also associated with sweat rate across these models (significant in two and trend-level in one). These findings are hypothesis-generating and may inform future mechanistic or interventional studies on lactic acid and palmar skin. Given the cross-sectional design, causality cannot be inferred.

## Introduction

Hands play essential roles in object manipulation, communication, and daily life, but they also serve as potential vectors for contact-mediated pathogen transmission.

The coronavirus disease 2019 (COVID-19) pandemic highlighted the role of hands in pathogen transmission and increased the frequency of hand hygiene practices, which can damage hand skin [1–4]. The pandemic also altered attitudes toward interpersonal touch [5–8].

Recent research has demonstrated that hands are not entirely defenseless in the context of infection transmission. For example, the skin on the volar side of the hand exhibits innate inactivation activity against bacteria and viruses [9,10], and there is a correlation between the strength of this inactivation activity and susceptibility to infections [11]. Although antimicrobial peptides are anti-pathogenic substances on the skin [12], lactic acid has also been identified as a component associated with this inactivation [11,13], highlighting its role in defense against infection.

This raises the question of whether the role of lactic acid on the volar hand skin is restricted to defense against infection. In dermatology, lactic acid is recognized as a type of natural moisturizing factor (NMF) that contributes to skin hydration [14–16]. For example, it is established that conditions characterized by dryness, such as atopic dermatitis, are associated with reduced lactic acid content [17]. However, these reports primarily focused on the forearms and cheeks. In contrast, for the palms, which significantly differ from these areas in terms of intercellular lipid content, stratum corneum thickness, and sweat gland density [18–20], the relationship between the amount of lactic acid and skin roughness is not well understood.

Understanding how palmar lactic acid relates to hand roughness and anti-pathogenic activity may inform future approaches to hand skin care and infection control. Therefore, this study examined associations among volar hand skin properties, palmar surface lactic acid content, and *in vitro* anti-pathogenic activity using partial correlation analysis that included other skin components and physiological factors.

## Materials and methods

### Study design

This study recruited participants from 15 December 2017–20 January 2018, and study visits and sampling were conducted from 25 January 2018–31 January 2018. The study involved healthy adult men and women aged 20–49 years, and no restrictions were imposed on participants’ usual use of skincare products (e.g., hand cream) prior to the measurements. A total of 54 participants were selected. Five individuals with failed skin image measurements were excluded, and the data from 49 participants were analyzed. All participants washed their hands with the same test soap under standardized conditions. The test soap was a surfactant-based formulation containing alkyl ether, carboxylic acid, alkyl ether sulfate, and alkyl glucoside, and did not contain any antimicrobial agents [21]. Participants rinsed their hands for 30 seconds with tap water and then for 10 seconds with purified water. To prevent contact with the evaluation area, they wore polyethylene mesh gloves (0950, SHOWA GLOVE Co.) for 2 hours to acclimate (20°C, 40% RH). After removal of the mesh gloves, participants acclimated for an additional 10 minutes before the measurements were started. S1 Fig shows the palmar measurement areas for hand scaling, *in vitro* anti-pathogenic activity, lactic acid content, and physiological properties. These measurements were conducted in defined, adjacent regions under standardized conditions, enabling integrated association analyses across chemical, physiological, and functional parameters.

### Hand skin scaling evaluation

Images of the palms were obtained using a flatbed scanner, and hand scaling was visually scored based on these images.

#### Acquisition of hand skin images.

In accordance with a previous report, images of the palmar side of the hand were obtained using the flatbed scanner (GT-X820; Seiko Epson Corporation) [22]. The images were captured at a resolution of 600 dpi with an 8-bit depth for each RGB channel.

#### Visual assessment of skin scaling.

Based on previously reported visual assessments of dry skin conditions and scaling-related signs [23,24], a hand scaling score was created by selecting representative images from all participants and classifying hand scaling into five levels based on the degree of scaling in the area shown in S1 Fig, panel a. Representative images for each score are shown in Fig 1. In this study, skin exhibiting scaling was defined as “hand dryness.” The evaluator (KN) assessed the degree of skin scaling within this area and was blinded to the results of the other factor analyses.

- Score 1: None (no signs of scaling).
- Score 2: Slight.
- Score 3: Moderate.
- Score 4: Marked (also present along wrinkles of the hands).
- Score 5: Severe (also exists in the shape of braiding between wrinkles).

**Fig 1:**
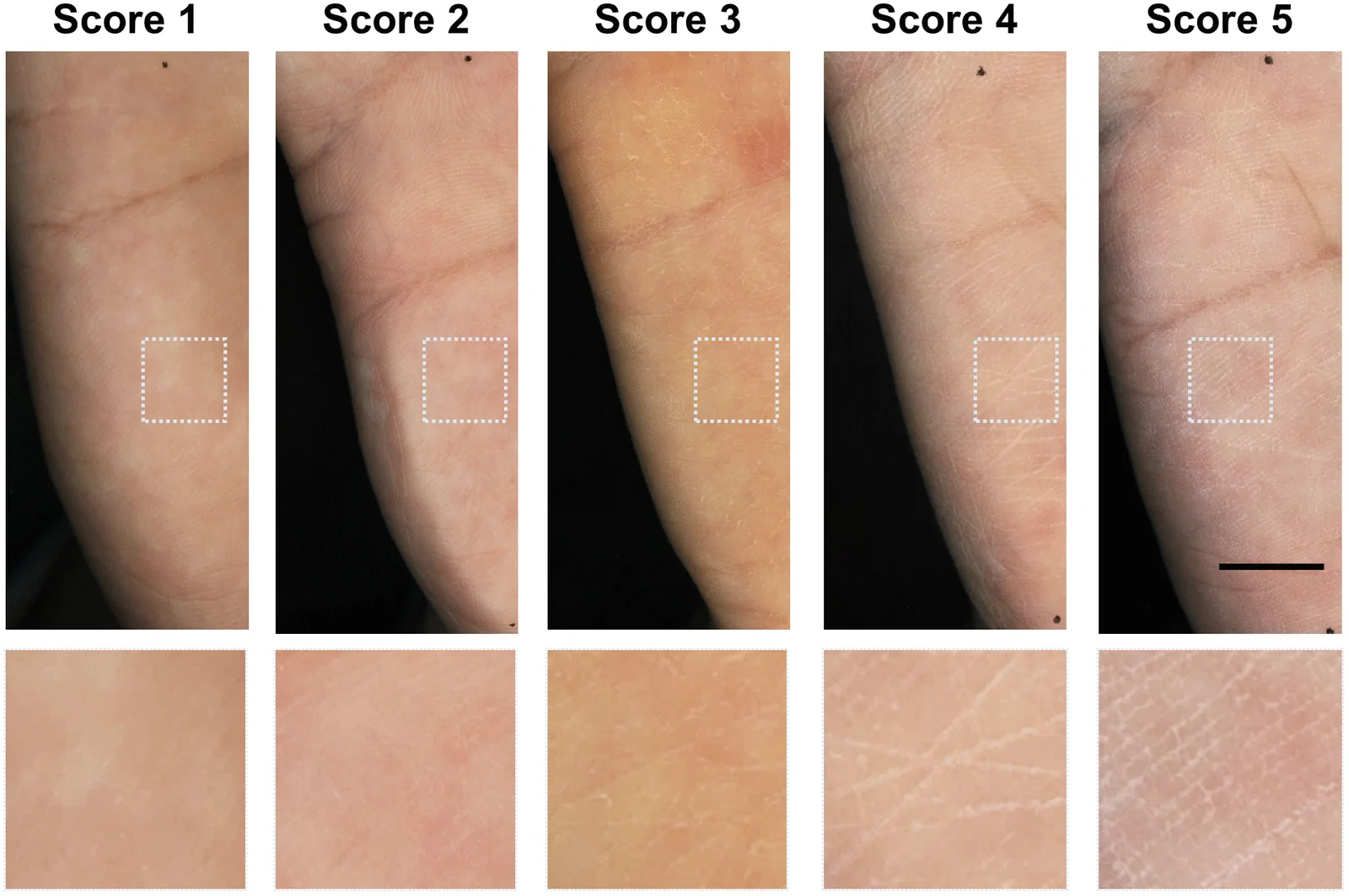
Representative palmar images defining hand scaling scores 1–5. Representative participants’ palmar images used to define the five-level visual hand-scaling score. From left to right, scores 1–5 (1 = none, 2 = slight, 3 = moderate, 4 = marked, 5 = severe). Dotted boxes indicate areas shown enlarged in the bottom row. Scale bar = 1 cm.

Additionally, participants (n = 5) with image areas that were difficult to analyze were excluded from the evaluation. Examples of such difficulties include the following: the skin adhered closely to the scanner surface, resulting in an image so flat that the texture could not be discerned. Vapor fogging, possibly due to sweat, obscured the visibility of the scaling.

### *In vitro* anti-pathogenic activity evaluation and lactic acid content of hand surface components

The procedure was conducted using the method described in a previous report [11]. A brief overview of the study is provided.

#### Bacterial strains and growth medium.

*Escherichia coli* NBRC3301 (*E. coli*) and *Staphylococcus aureus* NBRC13276 (*S. aureus*) were cultured in SCD and LB media, respectively. Bacterial growth was monitored to assess antibacterial activity using a Bio Microplate Reader (HiTS, Sinic Corporation).

#### Viral strain, cells, and growth medium.

Influenza virus A/Memphis/1/71 (H3N2) was cultured in Madin-Darby Canine Kidney (MDCK) cells with a serum-free medium. Viral titers were assessed using focus-forming assays in MDCK cells [25]. To evaluate antiviral activity, viral counts were determined by measuring the neuraminidase activity of the culture supernatant.

#### Evaluation of *in vitro* anti-pathogenic activity of hand surface components.

A 2.5 cm × 5 cm area of the hand (S1 Fig, panel b) was rubbed with a swab (1K1501; J.C.B. Industry Co.) soaked in 50% ethanol. The swab tips were placed in a microtube with 50% ethanol, and the solution was divided to test antibacterial and antiviral activities. The divided samples were then dried and dissolved in dimethyl sulfoxide and PBS. For antibacterial assessment, the sample solution was mixed at a ratio of 1:9 with bacteria suspended in purified water. For virus inactivation assessment, the sample solution was mixed at a ratio of 1:1 with virus suspended in purified water. Antibacterial activity was assessed by monitoring bacterial growth, and antiviral activity was assessed by measuring residual viral activity relative to the control. Thus, this assay evaluated anti-pathogenic activity under *in vitro* extract-based conditions.

#### Sample collection method for hand surface lactic acid analysis.

Lactic acid was collected by placing three round filter papers (2.1 cm in diameter; As One Corporation) on the palm (S1 Fig, panel c) and then applying 60 μL of ultrapure water to each one. The papers were left in place for 5 minutes before being retrieved. Thus, the measured values represent water-extractable palmar surface lactic acid content under these sampling conditions, rather than a direct surface concentration or lactic acid secretion rate.

#### Analysis of lactic acid.

Hand surface samples collected on three filter papers were desiccated. Following this, ultrapure water was added to the samples, and the supernatant was analyzed via liquid chromatography-mass spectrometry. The analysis was performed using an ACQUITY LC system (Waters) paired with an LTQ Orbitrap Velos Pro mass spectrometer (Thermo Fisher Scientific) and a Discovery HS F5 column (3-μm particle size, 2.1 mm internal diameter, 150 mm length; Sigma-Aldrich).

### Amounts of amino acids and ceramides in hand stratum corneum

Ceramides and amino acids were extracted from the stratum corneum and quantified by tape stripping using a solvent.

#### Sample collection and pretreatment.

For each participant, a palm surface area of 2.5 cm × 3 cm was stripped using acrylic adhesive film tape (456#40, Teraoka Seisakusho, Tokyo, Japan). The tape was dissolved in a chloroform/methanol/2-propanol solution (10:45:45). It was then filtered through glass wool and a portion of the filtrate was used for ceramide quantification. After extraction, a methanol/water (9:1) solution was added to the stratum corneum stripping tape and filtered through the same glass wool. The resulting filtrate was combined with the residual solution from ceramide analysis and dried. The dried material was subjected to liquid-liquid partitioning by adding chloroform and ultrapure water, and the aqueous phase was used to quantify amino acids as the NMF of the stratum corneum. Tetrahydrofuran was added to the extracted stratum corneum stripping tape, filtered through the same glass wool, and the filtrate was discarded. The glass wool was allowed to air dry, after which 6 mol/L hydrochloric acid was added, and the solution was hydrolyzed at 110°C for 24 hours. Subsequently, the hydrochloric acid was removed using a centrifugal evaporator, and the residue was dissolved in ultrapure water and used to quantify the amino acid content corresponding to the stratum corneum protein. The amounts of ceramides and stratum corneum amino acids were normalized to the amino acid content corresponding to stratum corneum protein and expressed as ng/μg-protein.

#### Ceramide and amino acid quantification methods.

Ceramides were analyzed using reverse-phase liquid chromatography-electrospray ionization mass spectrometry, as previously described [26]. For each sample, the relative ceramide class composition (% of total) was calculated as the amount of each class divided by the sum across all measured classes; detailed distributions are provided in S1 Table.

Amino acid analysis was performed using an ion-exchange column (#2622SC; Hitachi, Tokyo, Japan) and a Hitachi L-8800 amino acid analyzer as described previously [26].

### Physiological hand measurements

Skin pH, skin temperature, and sweat rate were assessed as described previously [13]. pH was determined using a multi-skin measurement device (MDD4) and a skin pH meter (pH 905; Courage + Khazaka Electronic GmbH). Skin temperature was recorded with a contact thermometer TM-300 (AS ONE Corp.). Sweat rate was evaluated using a micro sweat meter TPL3520 (TECHNO NEXT Co., Ltd.) [27].

### Ethical consideration

The study design and sample collection protocol received approval from the Institutional Review Board of Kao Corporation (approval no. S108-171024). All participants provided written informed consent following an explanation of the procedures. All experiments complied with the principles outlined in the Declaration of Helsinki.

### Statistical analyses

R version 4.2.1 (R Foundation, Vienna, Austria), with the psych, ppcor, corpcor, and ggraph packages installed, was used for association and statistical analyses. All associations between parameters were analyzed using Spearman’s rank correlation coefficients.

For the correlation and partial correlation coefficients between parameters, a non-correlation test was performed with multiplicity adjustment using the Benjamini–Hochberg procedure to control the false discovery rate (FDR). In the partial correlation analysis, all factors other than the two of interest were used as control variables. Partial correlations were used as descriptive measures of association and not as evidence of mediation; therefore, “associated” denotes statistical associations without implying causality. Statistical significance was defined as P < 0.05, whereas P < 0.1 was interpreted as a trend.

With N = 49 and a two-sided α = 0.05, the study has approximately 80% power to detect bivariate correlations of |ρ| ≈ 0.38 (Pearson approximation to Spearman). In the five-variable partial correlation models, each edge was conditioned on three covariates (k = 3; df = 44); the corresponding minimum detectable |ρ_p_| is ≈ 0.40, and the α = 0.05 critical value is ≈ 0.29.

## Results and discussion

### Correlation between hand scaling score and various factors (anti-pathogenic activity, lactic acid content, stratum corneum components, skin properties)

Fig 2 shows the distribution of scaling scores across all five categories (n per category = 5–16; total n = 49).

**Fig 2:**
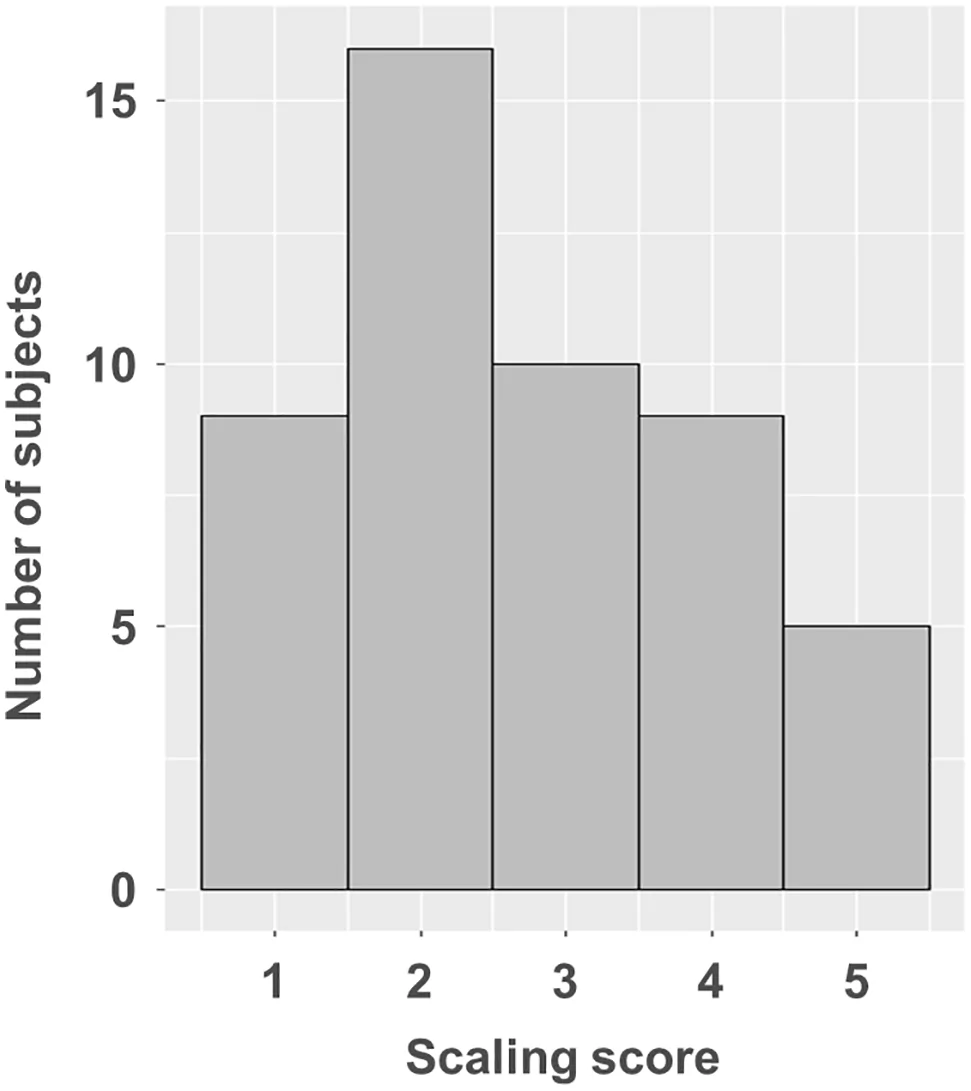
Histogram showing scaling score distribution of participants (n = 49).

Table 1 shows correlations of hand scaling score with anti-pathogenic activity, palmar surface lactic acid content, total amino acid content, total ceramide content, and skin physiological properties. The ceramide class composition (% of total ceramides, mean ± SD) and the total ceramide amount (ng/μg-protein, mean ± SD) are summarized in S1 Table. Although detailed class-level ceramide composition is provided for completeness, the present analyses focused on surface-associated factors rather than detailed epidermal lipid architecture. Additionally, the correlations between the hand scaling score and individual ceramide classes as well as amino acid groups are presented in Table 2. Fig 3 shows dot plots of lactic acid content and anti-pathogenic activity by hand scaling score. Because anti-pathogenic activity was expressed as log reduction, higher values indicate greater activity. From the univariate correlation coefficients and *P*-values in Table 1, the data indicate that hand scaling score was negatively associated with anti-pathogenic activity and lactic acid content. Additionally, as shown in Table 2, several ceramide classes (NS, ADS, and AS) were negatively correlated with the hand scaling score. While some ceramide classes showed associations with scaling in univariate analyses, our multivariable analyses focused on surface lactic acid and included total ceramide; class-level analyses of independence were beyond the scope of the present study.

**Table 1:** Correlations between scaling score and anti-pathogenic activities, lactic acid content, components of stratum corneum, and skin physiology.

	Scaling score	
	*ρ*	*P*-value^a^
**Influenza virus A** (log reduction)	−0.57	<0.001**
***E. coli*** (log reduction)	−0.41	0.011*
***S. aureus*** (log reduction)	−0.49	0.001**
**Lactic acid** (nmol/cm^2^)	−0.63	<0.001**
**Amino acids** (ng/μg-protein)	−0.16	0.379
**Ceramide** (ng/μg-protein)	−0.26	0.134
**Skin pH** (-)	0.25	0.150
**Temperature** (°C)	0.09	0.608
**Sweat rate** (mg/cm^2^/min)	−0.32	0.054

* *P* < 0.05, ** *P* < 0.01, a: adjusted by the FDR method.

**Table 2:** Correlations between scaling score and ceramide classes and amino acids.

		Scaling score	
		*ρ*	*P*-value^a^
Ceramide class (ng/μg-protein)	NDS	−0.17	0.269
	NS	−0.33	0.024*
	NH	−0.19	0.211
	NP	−0.05	0.728
	ADS	−0.34	0.021*
	AS	−0.45	0.002**
	AH	−0.20	0.193
	AP	−0.14	0.360
	EOS	−0.22	0.141
	EOH	−0.16	0.305
	EOP	−0.22	0.139
Amino acids (ng/μg-protein)	Asp	−0.12	0.416
	Thr	−0.10	0.525
	Ser	−0.15	0.330
	Glu	−0.05	0.765
	Pro	−0.24	0.118
	Gly	−0.15	0.317
	Ala	−0.21	0.163
	Cys	−0.22	0.153
	Val	−0.18	0.224
	Met	−0.14	0.342
	Ile	−0.20	0.193
	Leu	−0.19	0.224
	Tyr	−0.03	0.838
	Phe	−0.14	0.350
	Lys	−0.07	0.625
	His	−0.21	0.157
	Arg	−0.22	0.141

* *P* < 0.05, ** *P* < 0.01, a: adjusted by the FDR method.

**Fig 3:**
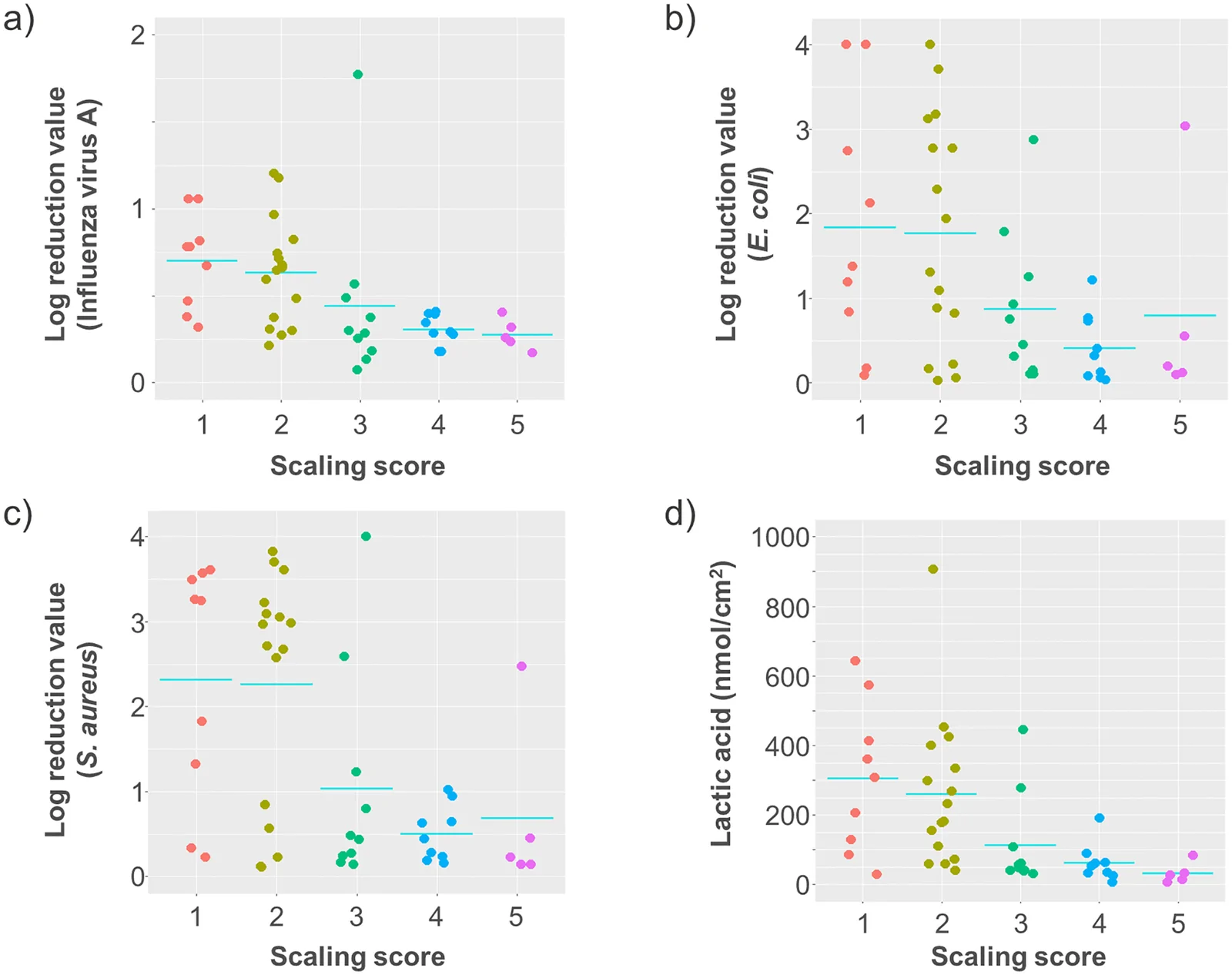
Scaling score vs palmar anti-pathogenic activity and lactic acid content. Anti-pathogenic activity against a) Influenza virus A, b) *E. coli*, and c) *S. aureus*, and d) palmar surface lactic acid content. The horizontal axis shows the hand scaling score (1–5). The vertical axis shows log_10_ reduction for panels a–c and lactic acid (nmol/cm²) for panel d. Horizontal cyan lines indicate the mean value within each scaling score category.

### Factors associated with hand dryness

The hand scaling score was inversely correlated with palmar surface lactic acid content (Table 1). Generally, dry skin is characterized by stratum corneum hyperproliferation [28], and skin with high hand scaling scores in this study may reflect a similar state. Excluding pathological cases, hyperproliferation may result from external irritant penetration and moisture loss due to reduced stratum corneum barrier function, which may involve decreased NMF, decreased intercellular lipids, and increased permeability [28–30].

To investigate factors associated with hand dryness, partial correlation analysis was conducted using five factors: hand scaling score, lactic acid content, stratum corneum amino acid content, ceramide content, and pH. The results are shown in Fig 4. Notably, skin conductance and capacitance were not measured because palmar skin exhibits abundant sweating, which makes it difficult to distinguish sweat-related signals from intrinsic hydration or barrier measurements.

**Fig 4:**
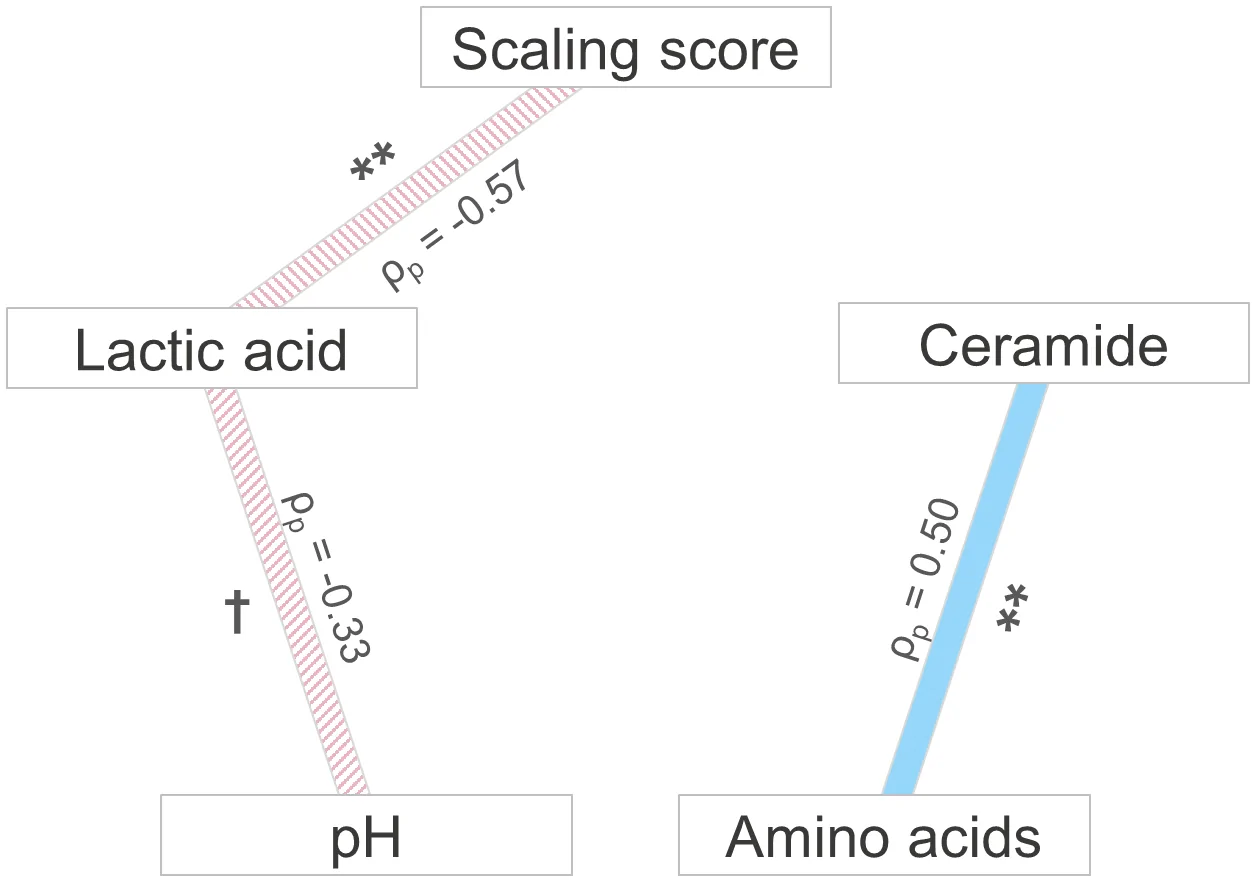
Partial correlation analysis of skin scaling-related factors. The selected factors were hand scaling score, lactic acid content, pH, amino acid content, and ceramide content. Lines indicate correlations with *P* < 0.1. Blue solid lines indicate positive correlations, and red hatched lines indicate negative correlations. † *P* < 0.1; * *P* < 0.05; ** *P* < 0.01.

Interestingly, stratum corneum amino acid and ceramide content, which are important indicators of healthy skin in the forearm and facial stratum corneum [31,32], did not significantly correlate with the hand scaling score, whereas surface lactic acid content showed a significant correlation. Within the scope of our data, surface lactic acid was more strongly associated with hand dryness than were stratum corneum amino acids or ceramides in the palm, a site that differs from the forearm in stratum corneum structure [18, 19]. Importantly, these observations should not be interpreted as negating the roles of amino acids or ceramides in epidermal hydration and barrier function; rather, the pattern may reflect the unique physiology of palmar skin and the use of scaling as the clinical endpoint.

### Factors associated with anti-pathogenic activity

Similarly, we considered factors related to anti-pathogenic activity. Uncharged non-dissociated lactic acid molecules penetrate pathogens, release protons, and exhibit inactivation effects [13]. This inactivation mechanism is more pronounced in 1) higher skin temperature ranges in which membrane fluidity increases, allowing lactic acid molecules to penetrate more easily, and 2) acidic conditions where the proportion of non-dissociated lactic acid molecules increases. As shown in Fig 5, anti-pathogenic activity was positively associated with lactic acid across all pathogens. By contrast, no clear association was observed with skin temperature, likely because the assays were conducted *in vitro* on skin extracts, which do not reflect *in vivo* skin temperature.

**Fig 5:**
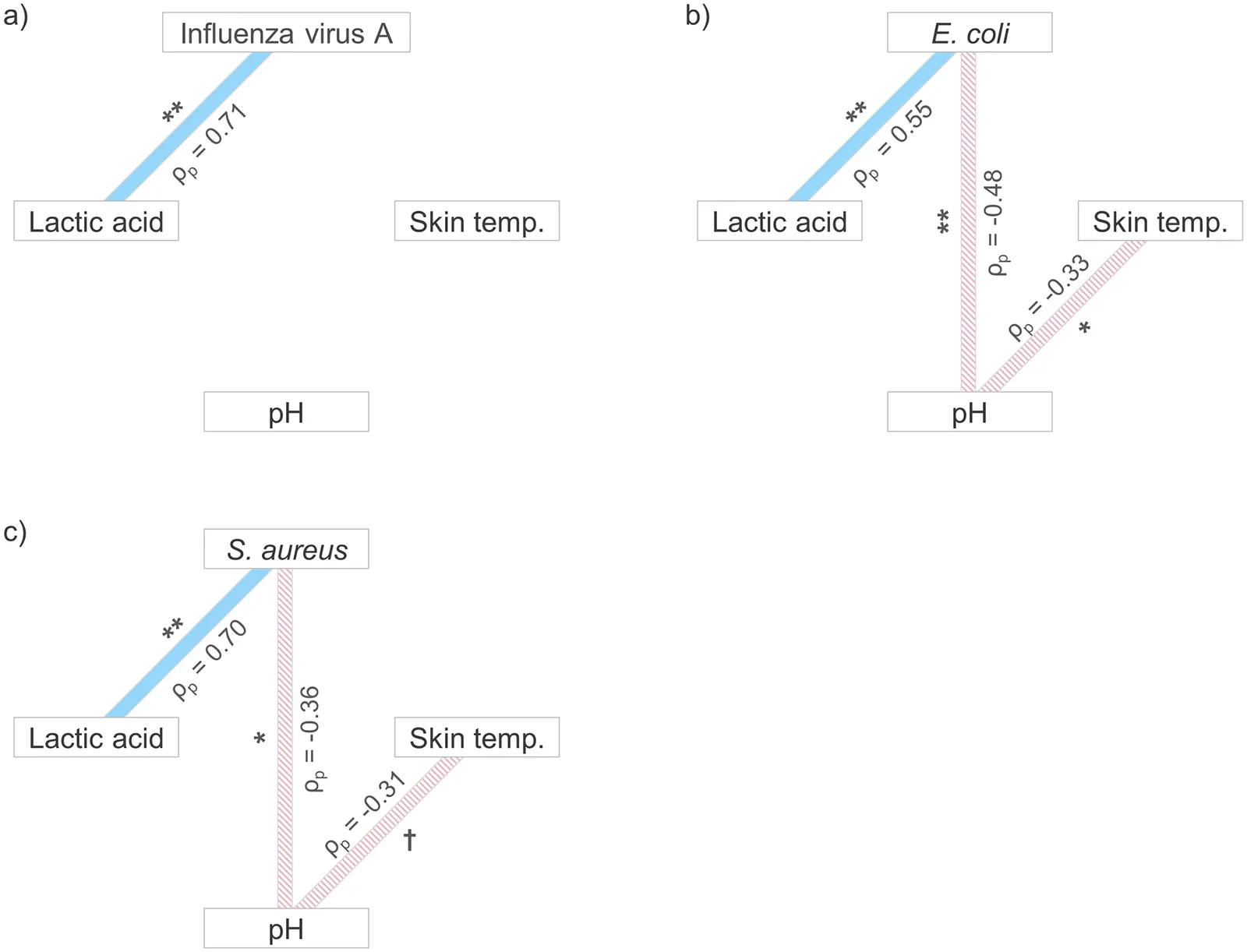
Partial correlation analysis of anti-pathogenic activity-related factors. Anti-pathogenic activity against a) Influenza virus A, b) *E. coli*, and c) *S. aureus*. The selected factors were anti-pathogenic activity, lactic acid content, pH, and skin temperature. Lines indicate correlations with *P* < 0.1. Blue solid lines indicate positive correlations, and red hatched lines indicate negative correlations. † *P* < 0.1; * *P* < 0.05; ** *P* < 0.01.

Regarding the association with pH, antibacterial activity against the two bacterial species showed negative correlations, as expected from the previously mentioned inactivation mechanism. In contrast, no correlation was observed between pH and antiviral activity. This may be due to the degree of PBS dilution used in the inactivation evaluation. The skin extract was dissolved in dimethyl sulfoxide and PBS; however, for antibacterial evaluation, it was mixed with the bacterial suspension in a 1:9 ratio, whereas for viral inactivation evaluation, it was mixed with the viral suspension in a 1:1 ratio. Therefore, in the viral inactivation evaluation with a higher PBS proportion, the buffering action of PBS may have prevented the pH of the skin extract from being fully reflected.

### Factors associated with lactic acid content

The factors related to lactic acid content were subsequently considered. Because lactic acid in the stratum corneum is primarily secreted by sweat glands [33], we first selected sweat rate as a candidate factor. Additionally, skin temperature was included as a candidate factor contributing to sweating [34]. Furthermore, pH was selected as a factor reflecting the quality of sweat, with the amino acids in the stratum corneum as factors related to NMF. The results of the partial correlation analysis for lactic acid and the candidate factors are shown in Fig 6. As shown in Fig 6, lactic acid content was associated with sweat rate and pH.

**Fig 6:**
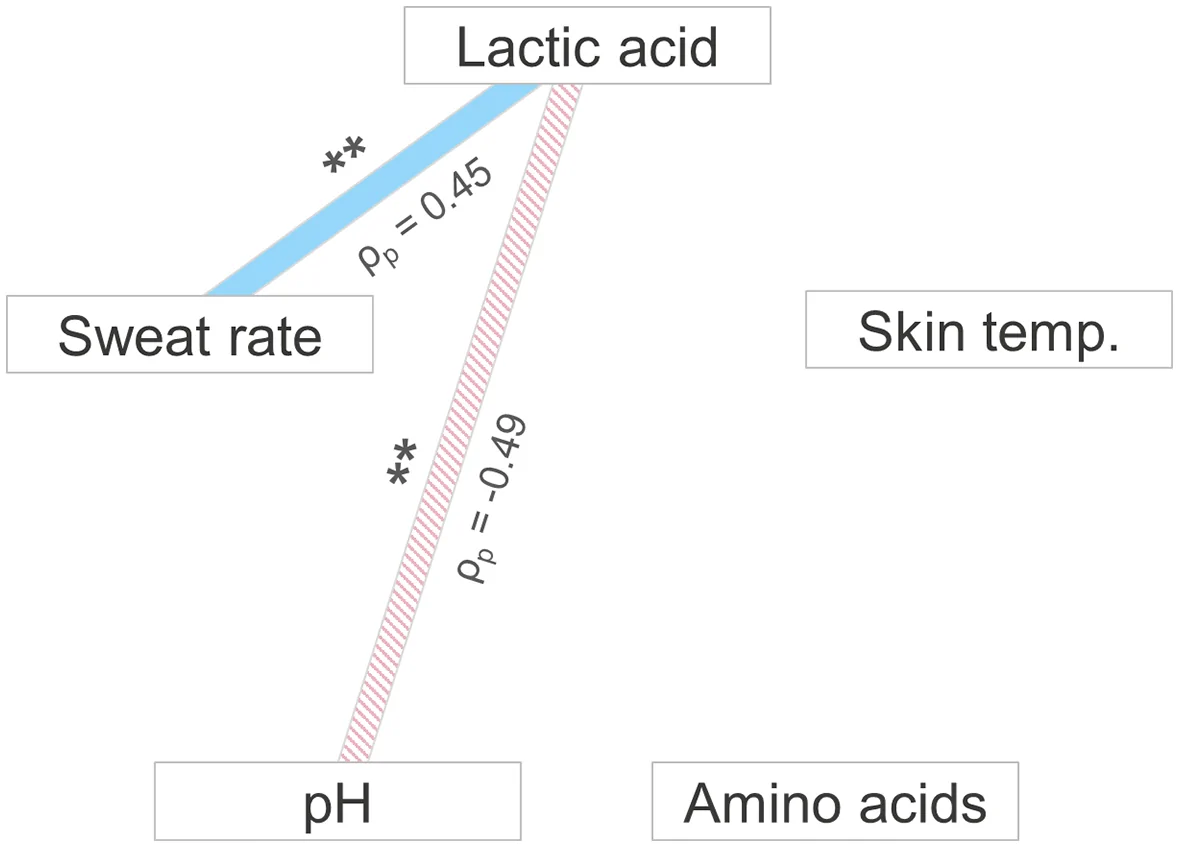
Partial correlation analysis of lactic acid-related factors. The selected factors were lactic acid content, sweat rate, pH, amino acid content, and skin temperature. Lines indicate correlations with *P* < 0.1. Blue solid lines indicate positive correlations, and red hatched lines indicate negative correlations. † *P* < 0.1; * *P* < 0.05; ** *P* < 0.01.

### Analysis of the relationship between hand dryness, anti-pathogenic activity, and lactic acid content

Figs 4–6 indicated that sweat rate and pH should also be considered, in addition to hand scaling score, anti-pathogenic activity, and lactic acid content. The results of the partial correlation analysis using these five factors are shown in Fig 7.

**Fig 7:**
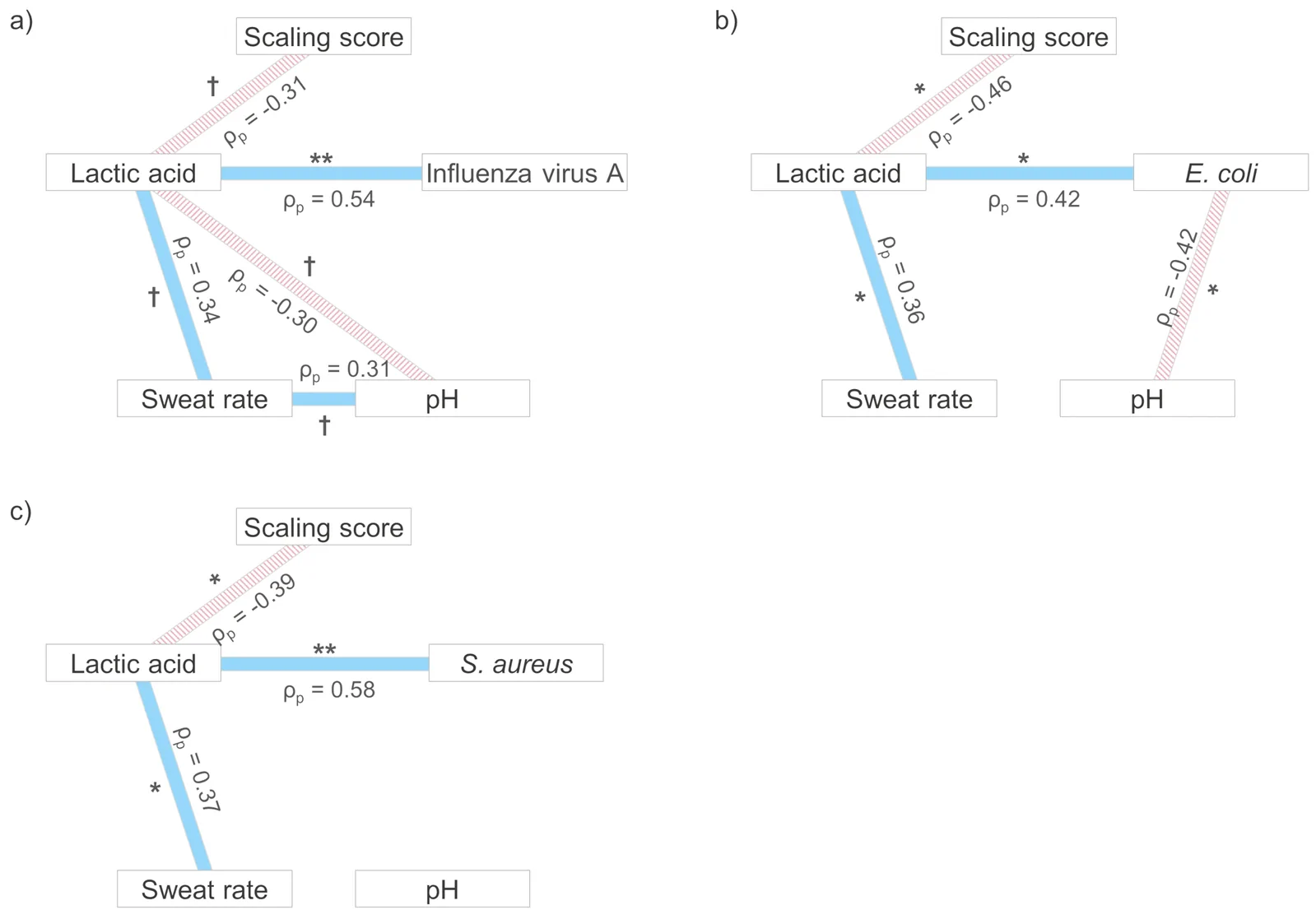
Partial correlation analysis of scaling score, anti-pathogenic activity, and lactic acid-related factors. Anti-pathogenic activity against a) Influenza virus A, b) *E. coli*, and c) *S. aureus*. The selected factors were scaling score, anti-pathogenic activity, lactic acid content, sweat rate, and pH. Lines indicate correlations with *P* < 0.1. Blue solid lines indicate positive correlations, and red hatched lines indicate negative correlations. † *P* < 0.1; * *P* < 0.05; ** *P* < 0.01.

Although Table 1 shows significant simple correlations between anti-pathogenic activity and hand scaling score, these associations were attenuated to non-significance after adjustment for lactic acid, sweat rate, and pH (Fig 7a–7c). In the same models, lactic acid was associated with both greater *in vitro* anti-pathogenic activity and lower scaling, suggesting that lactic acid may be a shared correlate; however, alternative explanations, including residual confounding, remain possible. These findings are consistent with prior *in vitro* evidence of the antimicrobial activity of lactic acid.

Furthermore, surface lactic acid content was positively associated with sweat rate, showing a trend in Fig 7a and significant correlations in Fig 7b and 7c. Because lactic acid on the skin surface is considered to primarily originate from sweat glands, this association is biologically plausible. Palmar sweat has thermoregulatory and grip-related functions [34–37]. Our findings suggest that palmar sweat may also contribute to surface lactic acid content, with possible relevance to hand skin condition and *in vitro* anti-pathogenic activity. These potential implications for hand dryness and contact transmission merit further study.

The positive trend between sweat rate and pH (Fig 7a) suggests that increased sweating may raise pH, potentially reducing the inactivation effect of lactic acid. Because lactic acid concentration in sweat varies widely and excessive sweating can produce sweat with lower lactic acid concentration and higher pH [34,38,39], both surface lactic acid content and palmar pH may be relevant targets for future controlled studies.

### Limitations

This study has some limitations. Its cross-sectional design precludes causal inference and formal mediation testing. Residual confounding by unmeasured factors cannot be excluded, including recent use of moisturizers or other topical products, environmental and occupational exposures, habitual hand hygiene practices, underlying dermatologic conditions, and skin microbiome composition. These factors may influence skin pH, scaling, palmar surface lactic acid content, and *in vitro* anti-pathogenic activity. In addition, hand scaling was assessed by visual scoring by a single evaluator, which may introduce subjective measurement bias despite blinding to other measurements. Although the scoring approach was based on previously reported visual assessments of dry skin conditions and scaling-related signs, intra-rater reliability was not formally evaluated in this study. The filter-paper extraction method using ultrapure water may collect water-soluble components from multiple sources, including sweat-derived components, surface residues, and compounds extracted from or diffusing from the superficial stratum corneum. Therefore, the measured lactic acid values should be interpreted as water-extractable palmar surface lactic acid content under the sampling conditions, rather than as true surface concentration or dynamic secretion rate. The anti-pathogenic assay was performed under *in vitro* extract-based conditions after ethanol extraction, drying, and reconstitution in dimethyl sulfoxide and PBS. These procedures may alter pH, metabolite stability, and physiological relevance compared with the native skin surface; therefore, the measured activity should be interpreted as the anti-pathogenic activity of extracted components rather than the full *in vivo* function of the skin surface. Thus, prospective or interventional studies are needed to determine whether modifying palmar lactic acid or pH leads to clinically meaningful changes in hand skin condition or transmission risk. Ultimately, these associations do not establish causality or mediation and should be interpreted as hypothesis-generating.

## Conclusion

This study investigated associations among hand scaling, *in vitro* anti-pathogenic activity, palmar surface lactic acid content, stratum corneum components, and skin physiological properties. The main findings were as follows: 1) lower scaling was associated with greater *in vitro* anti-pathogenic activity; 2) palmar surface lactic acid content was the factor most strongly associated with both lower scaling and greater *in vitro* anti-pathogenic activity; and 3) sweat-derived lactic acid and palmar pH may be relevant to hand skin condition and pathogen survival on the skin.

These hypothesis-generating findings suggest that palmar surface lactic acid may be related to both hand scaling and *in vitro* anti-pathogenic activity, and warrant confirmation in prospective or interventional studies.

## Supporting information

S1 Fig Measurement areas of scaling, *in vitro* anti-pathogenic activity, lactic acid content, physiological properties, and components of stratum corneum. (a) Hand skin scaling, (b) *in vitro* anti-pathogenic activity, (c) lactic acid content, (d) skin pH, (e) skin temperature, (f) sweat rate, and (g) concentration of amino acids and ceramides.
(TIF)

S1 Table Ceramide class composition of palmar stratum corneum (n = 49). SD, standard deviation. The last row reports total ceramide (ng/μg-protein). Percentages may not sum to 100% due to rounding.
(DOCX)

S1 Dataset De-identified participant-level dataset used for all analyses. (CSV)

S1 Code R script that computes Benjamini–Hochberg FDR adjusted Spearman correlations and saves the results to “[Result]correlation.txt”. (TXT)

S2 Code R script that computes Spearman partial correlations (ppcor), applies Benjamini–Hochberg FDR to *P*-values, and exports coefficients, *P*-values, and statistics to “[Result]partial_correlation.txt”. (TXT)

S3 Code R script that computes approximate minimum detectable correlations (80% power) and α = 0.05 critical correlation values for bivariate and partial (k = 3) correlations using Fisher’s z approximation (outputs to console). (TXT)
