# Supplementary material for: Associations of palmar surface lactic acid with hand scaling and in vitro pathogen inactivation: A cross-sectional study
Source: PLoS One. 2026 Nov 10;21(11):e0362451. doi: 10.1371/journal.pone.0362451 (PMC13750577; doi:10.1371/journal.pone.0362451)
Supplement: Supplementary file 1 [file pone.0362451.s002.docx]

**S1 Table. Ceramide class composition of palmar stratum corneum (n = 49).**

| Ceramide class | % of total (mean ± SD) |
| --- | --- |
| NDS | 9.2 ± 1.4 |
| NS | 15.4 ± 2.1 |
| NH | 18.9 ± 1.7 |
| NP | 12.2 ± 3.8 |
| ADS | 3.9 ± 0.7 |
| AS | 11.4 ± 1.6 |
| AH | 12.6 ± 1.7 |
| AP | 9.8 ± 1.8 |
| EOS | 2.5 ± 0.8 |
| EOH | 2.8 ± 1.0 |
| EOP | 1.4 ± 0.7 |
| Total ceramide (ng/μg-protein, mean ± SD) | 5.3 ± 1.8 |

SD, standard deviation. The last row reports total ceramide (ng/μg‑protein). Percentages may not sum to 100% due to rounding.
