# Supplementary material for: Associations of palmar surface lactic acid with hand scaling and in vitro pathogen inactivation: A cross-sectional study
Source: PLoS One. 2026 Nov 10;21(11):e0362451. doi: 10.1371/journal.pone.0362451 (PMC13750577; doi:10.1371/journal.pone.0362451)
Supplement: Supplementary file 2 [file pone.0362451.s007.docx]

**Response to the Academic Editor and Reviewers**

Manuscript ID: PONE-D-26-03354

Title: Associations of palmar surface lactic acid with hand scaling and in vitro pathogen inactivation: a cross-sectional study

We thank the Editor and the journal staff for their careful review of our manuscript and for the opportunity to revise it. We have revised the manuscript and Supporting Information files in accordance with the journal requirements and reviewer comments. The main changes are summarized below.

**Response to the Academic Editor**

**Journal Requirement 1: PLOS ONE style requirements**

We reviewed the manuscript and Supporting Information files to ensure compliance with PLOS ONE style requirements. We revised the manuscript to improve conciseness, standardize terminology, and correct minor grammatical and typographical issues. We also revised the Supporting Information file names to make them clearer and more consistent.

For example, “S1Fig.tif” was renamed to “S1_Fig.tif”. Similar file-name revisions were made for the other Supporting Information files.

**Journal Requirement 2: Competing Interests statement**

We have updated the Competing Interests statement as requested and included the revised statement in the cover letter.

Updated Competing Interests statement:

“All authors are employees of Kao Corporation, a commercial company. There are no products in development or marketed products to declare. This does not alter our adherence to PLOS ONE policies on sharing data and materials.”

**Journal Requirement 3: Anonymization of S1 Dataset**

To prevent sharing potentially identifying data or hidden Excel content, we reviewed the participant-level dataset and replaced the previously submitted S1Dataset.xlsx file with S1_Dataset.csv, a fully anonymized plain UTF-8 CSV file. The revised dataset contains only anonymized sequential participant IDs and study variables used for the analyses, including scaling scores; in vitro anti-pathogenic log_10_ reductions for influenza A/H3N2, E. coli, and S. aureus; palmar surface lactic acid; skin pH and temperature; sweat rate; stratum corneum amino acids; and stratum corneum ceramides. We confirm that the revised CSV file does not include names, initials, contact information, specific dates, location data, detailed ages, or other direct or indirect identifiers.

**Response to Reviewer #1**

**Comment:**

The manuscript of the research paper is nicely written, however, lacks the precision so the manuscript became very lengthy. Therefore, the manuscript should be abbreviated and need to be written in short.

**Response:**

We thank the reviewer for this constructive comment. We revised the manuscript to improve precision and conciseness. Specifically, we shortened background statements in the Introduction, reduced general explanatory text in the Discussion, removed redundant descriptions around the partial-correlation results, and streamlined figure captions. We also corrected minor grammatical and typographical issues throughout the manuscript. These revisions were made without changing the study design, analyses, results, or conclusions.

**Response to Reviewer #2**

**Comment 1:**

Some of the confounding factors like use of moisturizers and topical products, environmental exposure, occupational exposure, and hand hygiene has direct influence on skin pH, scaling. Microbiome, and other bioactive products such as lactic acid production level. Therefore, these factors may be included as a covariate or acknowledge as major confounder, also sensitivity analysis excluding heavy product users may be considered.

**Response:**

We thank the reviewer for this important comment. We agree that moisturizers and other topical products, environmental and occupational exposures, habitual hand hygiene practices, and skin microbiome composition may influence skin pH, scaling, palmar surface lactic acid content, and in vitro anti-pathogenic activity. Because detailed information on these factors was not available for all participants, we could not include them as covariates or perform a sensitivity analysis excluding heavy topical-product users. We have therefore revised the Limitations section to acknowledge these factors as potential unmeasured confounders.

**Comment 2:**

The hand scaling is measured as visual scoring which might introduce subjectivity measurement bias. Hence intra-rater reliability may be reported, or the method validity may be justified. Also, it may be highlighted as the limitation explicitly.

**Response:**

We thank the reviewer for this important comment. We agree that visual scoring may introduce subjective measurement bias. We have revised the Methods section to clarify that the hand scaling score was developed based on previously reported visual assessments of dry skin conditions and scaling-related signs. The evaluator was blinded to the other measurements to reduce potential bias. However, intra-rater reliability was not formally evaluated in this study. We have therefore explicitly acknowledged the potential subjectivity of visual scoring and the lack of formal intra-rater reliability assessment in the Limitations section.

**Comment 3:**

In the methodology section, the filter paper extraction method using ultrapure water may dilute endogenous metabolites and capture a mixture of sweat-derived components, surface contaminants, and stratum corneum diffusion products. As a result, it is unclear whether the measured lactic acid reflects true surface concentration or dynamic sweat secretion. The authors should clarify whether the values represent steady-state levels or secretion rates and more explicitly discuss the methodological limitations of this sampling approach.

**Response:**

We thank the reviewer for this important comment. We agree that the filter-paper extraction method using ultrapure water does not directly measure the native surface concentration or secretion rate of lactic acid. Rather, the method recovers water-soluble lactic acid under the sampling conditions, which may include sweat-derived components, surface residues, and compounds extracted or diffusing from the superficial stratum corneum. We have clarified in the Methods section that the measured values represent water-extractable palmar surface lactic acid content, rather than a direct surface concentration or lactic acid secretion rate. We have also added this methodological limitation to the Limitations section.

**Comment 4:**

Line 93-94, the soap composition may influence the residual skin chemistry, if possible, the content of soap used may be highlighted to have more clearer view in term of the chemical interaction if any.

**Response:**

We thank the reviewer for this helpful comment. We agree that the soap composition may influence residual skin chemistry and potential chemical interactions on the skin surface. We have revised the Methods section to clarify that all participants used the same test soap under standardized washing conditions. The test soap was a surfactant-based formulation containing alkyl ether, carboxylic acid, alkyl ether sulfate, and alkyl glucoside, and did not contain any antimicrobial agents. We also specified the rinsing procedure to make clear that the washing protocol was standardized across all participants.

**Comment 5:**

Line 159-162: The ethanol extraction, drying, and reconstitution in dimethyl sulfoxide (DMSO)/PBS may substantially alter pH, metabolite stability, and overall physiological relevance of the samples. Consequently, the assay reflects antimicrobial activity of extracted components rather than the true in-vivo skin surface function. Therefore, it is suggested authors should explicitly state that the results represent antimicrobial activity under in vitro extract-based conditions.

**Response:**

We thank the reviewer for this important comment. We agree that ethanol extraction, drying, and reconstitution in dimethyl sulfoxide/PBS may alter pH, metabolite stability, and physiological relevance compared with the native skin surface. We have clarified that the assay evaluated anti-pathogenic activity under in vitro extract-based conditions. We have also added this point to the Limitations section, noting that the measured activity should be interpreted as the anti-pathogenic activity of extracted components rather than the full in vivo function of the skin surface.

**Comment 6:**

Some of the minor grammatical, scientific notation concern may be given to improve the draft.

**Response:**

We thank the reviewer for this comment. We carefully reviewed the manuscript and corrected minor grammatical, typographical, and scientific notation issues throughout the text. In particular, we standardized terminology such as “anti-pathogenic activity,” corrected notation and unit formatting, and revised several sentences for clarity and conciseness.
